# The feasibility of a novel limited field of view spiral cine DENSE sequence to assess myocardial strain in dilated cardiomyopathy

**DOI:** 10.1007/s10334-019-00735-5

**Published:** 2019-01-29

**Authors:** Upasana Tayal, Ricardo Wage, Pedro Filipe Ferreira, Sonia Nielles-Vallespin, Frederick Howard Epstein, Daniel Auger, Xiaodong Zhong, Dudley John Pennell, David Nigel Firmin, Andrew David Scott, Sanjay Kumar Prasad

**Affiliations:** 1 National Heart Lung Institute, Imperial College London, London, UK; 2 Cardiovascular Magnetic Resonance Unit, Royal Brompton Hospital, London, SW3 6NP UK; 3 National Heart, Lung and Blood Institute, National Institutes of Health, Bethesda, MD USA; 4 Biomedical Engineering, University of Virginia, Charlottesville, VA USA; 5 Siemens Healthcare, Atlanta, GA USA

**Keywords:** Strain, Dilated cardiomyopathy, DENSE, Cardiovascular magnetic resonance, Function, Text

## Abstract

**Objective:**

Develop an accelerated cine displacement encoding with stimulated echoes (DENSE) cardiovascular magnetic resonance (CMR) sequence to enable clinically feasible myocardial strain evaluation in patients with dilated cardiomyopathy (DCM).

**Materials and methods:**

A spiral cine DENSE sequence was modified by limiting the field of view in two dimensions using in-plane slice-selective pulses in the stimulated echo. This reduced breath hold duration from 20RR to 14RR intervals. Following phantom and pilot studies, the feasibility of the sequence to assess peak radial, circumferential, and longitudinal strain was tested in control subjects (*n* = 18) and then applied in DCM patients (*n* = 29).

**Results:**

DENSE acquisition was possible in all participants. Elements of the data were not analysable in 1 control (6%) and 4 DCM r(14%) subjects due to off-resonance or susceptibility artefacts and low signal-to-noise ratio. Peak radial, circumferential, short-axis contour strain and longitudinal strain was reduced in DCM patients (*p* < 0.001 vs. controls) and strain measurements correlated with left ventricular ejection fraction (with circumferential strain *r* = − 0.79, *p* < 0.0001; with vertical long-axis strain *r* = − 0.76, *p* < 0.0001). All strain measurements had good inter-observer agreement (ICC > 0.80), except peak radial strain.

**Discussion:**

We demonstrate the feasibility of CMR strain assessment in healthy controls and DCM patients using an accelerated cine DENSE technique. This may facilitate integration of strain assessment into routine CMR studies.

**Electronic supplementary material:**

The online version of this article (10.1007/s10334-019-00735-5) contains supplementary material, which is available to authorized users.

## Introduction

In cardiomyopathy patients, early disease detection and sensitive markers of disease progression are important. Left ventricular ejection fraction (LVEF) is the mainstay to assess myocardial function, but has a number of limitations [1]. It is relatively insensitive and compensatory changes in global circumferential strain as well as in wall thickening can mask important disease [2].

Assessment of myocardial strain complements assessment of myocardial function. It affords the opportunity for more sensitive detection of disease and overcomes some of the limitations of myocardial geometry. In addition, myocardial strain assessment, particularly longitudinal strain, is prognostically important in dilated cardiomyopathy (DCM) [3] and can detect early LV dysfunction [4]. With a high genetic predisposition in DCM, early recognition and therapeutic intervention may facilitate timely disease modifying therapy.

Cardiovascular magnetic resonance (CMR) is becoming increasingly important in DCM due to advanced tissue characterization [5, 6], but the CMR assessment of myocardial strain has been limited. CMR tissue tagging [7, 8] is the most studied method, but its application has been hindered by tag fade in diastole, low spatial resolution, and complex post-processing, reflected by low utilisation [9].

There is, therefore, a need for a robust CMR methodology to assess strain in DCM patients. Displacement encoding with stimulated echoes (DENSE) [10–12] is an established technique to measure myocardial strain, but its clinical application has been limited by long breath hold durations or navigator gating which can be impractical and time consuming, particularly for patients with heart failure [13]. We, therefore, developed an accelerated cine DENSE sequence to enable the evaluation of strain in a breath hold time suitable for patients with DCM.

In this paper, we present the results of a feasibility study evaluating the CMR acquisition and analysis of myocardial strain using a novel cine DENSE sequence, first in healthy control participants and then applied to a cohort of DCM patients.

## Materials and methods

This study was approved by the regional research ethics committees and all participants gave written informed consent. CMR was performed at 3T (Siemens Skyra, Erlangen, Germany) using an anterior body and spine receive coil array.

### Sequence modification

**Fig. 1:**
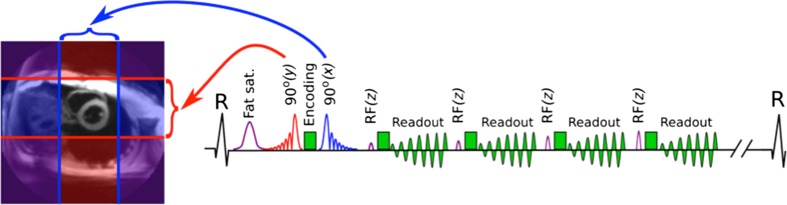
Novel accelerated zonal spiral cine DENSE sequence. The first RF pulse (red) reduces the y FOV to the region between the red lines, while the second RF pulse (blue) reduces the perpendicular FOV, leaving a region around the heart to image. The schematic sequence diagram also shows the R wave of the ECG, fat suppression (fat sat.), the DENSE encoding and spiral gradients (green) and the slice-selective RF pulses (purple) with increasing flip angle used for imaging. In our pilot study, a mean of 27 frames were acquired (30 ms each), which equates to 54 spiral readouts in each cardiac cycle (TR = 15 ms)

We modified a cine DENSE sequence with a spiral k-space trajectory [14] by reducing the imaged field of view (FOV) to a small square region around the left ventricle while avoiding aliasing artefacts (Fig. 1). This was achieved via perpendicular in-plane slice selection gradients applied to the first and second of the three radiofrequency (RF) pulses required to produce the stimulated echo. The smaller FOV means that fewer spiral interleaves can be used for a given spatial resolution without undersampling. To provide a satisfactory excitation profile, with minimal additional echo time, a time-reversed pair of asymmetric RF pulses (81% and 19% asymmetry) was used.

### Phantom study

The in-plane excitation profile and signal-to-noise ratio (SNR) of the proposed method was assessed in an 11 cm diameter cylindrical phantom filled with 40 g/L agar in tap water. T1 and T2 were measured at 1060 ± 20 ms and 58.1 ± 0.7 ms, respectively.

The in-plane excitation profile was assessed by acquiring DENSE data with a stimulated echo FOV of 50 × 50 mm^2^ (determined by the slice selection of the first two RF pulses in Fig. 1) and a larger readout FOV of 250 × 250 mm^2^.

To determine the maximum size of the stimulated echo FOV for a given readout FOV without aliasing artefacts DENSE imaging was performed using a 70 × 70 mm^2^ readout FOV and a stimulated echo FOV of 70 × 70 mm^2^, 90 × 90 mm^2^ and 110 × 110 mm^2^.

To determine the effect of the reduced FOV technique on the magnitude image SNR, DENSE acquisitions were performed with 4 protocols, each repeated 20 times to provide noise estimates [15]. Three protocols were those used in the pilot healthy volunteer study below (20RR intervals, 360 × 360 mm^2^ FOV; 14RR-intervals, 224 × 224 mm^2^ FOV; and 8RR-intervals, 120 × 120 mm^2^ FOV) and the fourth was identical to the 20RR-interval acquisition without the reduced FOV technique. Single image SNR was calculated using the multiple repetitions method [15] and a mean ± standard deviation was calculated for an artefact-free region within the phantom.

### Pilot study

A pilot study (originally reported in [16]) evaluated the performance of this novel sequence in vivo with increasingly short breath hold durations. Eight healthy volunteers were imaged using the novel reduced FOV sequence with three different FOVs and breath hold durations.

DENSE was performed in a mid-ventricular short-axis slice using 3.2 × 3.2 mm^2^ acquired spatial resolution, 8 mm slice thickness, 30 ms temporal resolution, 2 spiral interleaves per RR-interval, 2-directional encoding at 0.06 cycles/mm, chemical shift selective fat saturation, repetition time/echo time (TR/TE) 15/1.0 ms, and variable flip angle (maximum of 20°). Unwanted echo pathways were minimised via CSPAMM-like encoding and through-plane dephasing (0.08 cycles/mm). Second-order B0 shimming was performed using a cardiac specific method [17]. As in the phantom SNR study, the three protocols were run with FOVs of 360 × 360 mm^2^, 224 × 224 mm^2^ and 120 × 120 mm^2^ (equal stimulated echo and readout FOV) with corresponding breath hold durations of 20RR intervals, 14RR intervals, and 8RR intervals. The reduction in FOV was achieved via a reduction in the total number of spiral interleaves from 8, to 6, to 4.

### Study cohort

#### Healthy control cohort

The healthy control cohort (*n* = 18) comprised individuals who did not have a history of medical illness, were not taking regular medication, and did not have evidence of cardiac structural or functional impairment on CMR.

#### DCM cohort

The DCM cohort was prospectively recruited (*n* = 29). Inclusion criteria were meeting criteria for a diagnosis of DCM (Supplementary materials), over 18 years of age, sinus rhythm, and the absence of a contraindication to CMR.

### CMR acquisition

All participants underwent CMR for assessment of cardiac structure, function, and myocardial strain.

#### Acquisition and assessment of cardiac structure and function

End-expiratory breath hold balanced steady-state free precession (bSSFP) cine images were acquired in the 3 long-axis planes (horizontal long axis, HLA; right ventricular outflow tract, RVOT; left ventricular outflow tract, LVOT); and 8 mm short-axis slices (2 mm gap) from the atrioventricular ring to the apex as previously described [5] (acquisition parameters in Supplementary materials). Biventricular volumes, function, and left ventricular (LV) mass were measured using a semi-automated threshold-based technique (CMRtools, Cardiovascular Imaging Solutions, London, UK). Left ventricular wall thickness and left atrial area were assessed as outlined in Supplementary materials. All volume and mass measurements were indexed to body surface area and referenced to age and gender [18].

#### Acquisition and assessment of myocardial strain data using modified cine spiral DENSE sequence

Based on the initial pilot study, DENSE acquisitions were performed using the 14RR interval protocol (224 × 224 mm^2^ FOV, readout and stimulated echo FOV equal) described above. Images were acquired at the mid-ventricular short-axis (SAX) level and in 2 long-axis planes (horizontal, HLA and vertical, VLA) using the novel reduced FOV sequence and an optimised protocol. All images were reconstructed online at the scanner. Comparative images were not acquired with a breath hold duration of 20 RR intervals, as these data were acquired within a longer protocol. We did not wish to subject patients with dilated cardiomyopathy to multiple long breath holds that they would find difficult and potentially lead to aborted studies.

### DENSE analysis

**Fig. 2:**
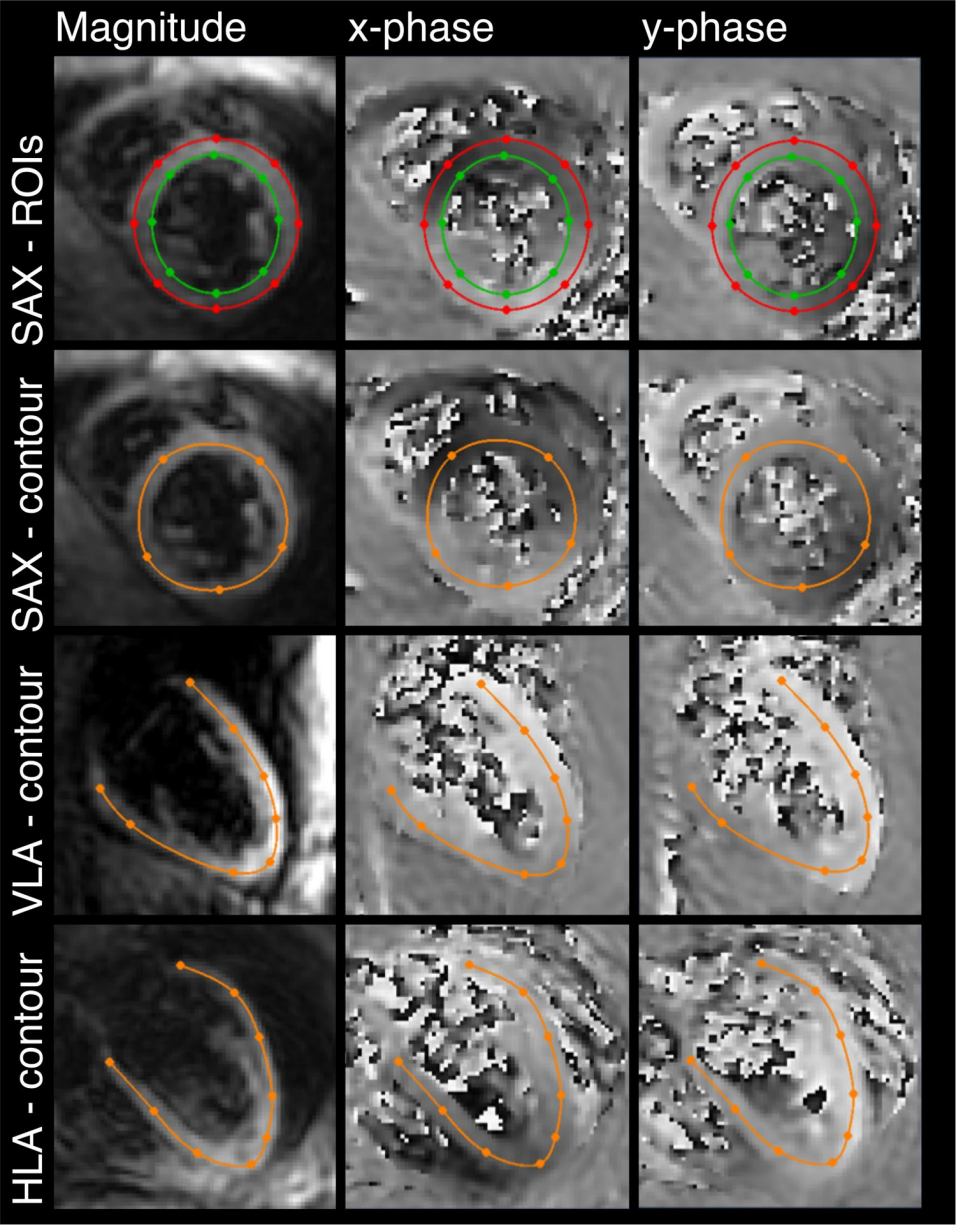
Example regions of interest for the assessment of strain. Top line shows short-axis (SAX) image with endo-epicardial contour for assessment of radial and circumferential strain. Lower panels show SAX, vertical long-axis (VLA), and horizontal long-axis (HLA) image with contour lines shown in orange for assessment of contour strain. The left-hand images in each panel are magnitude images and the two right-hand images are phase images, where the displacement of each pixel is encoded in the image phase (*x* and *y* directions). The SAX images are cropped to a FOV of 110 × 100 mm^2^ and the long-axis images are cropped to a FOV of 130 × 130 mm^2^

Images were analysed and strain extracted from the DENSE data using semi-automated MATLAB (The Mathworks, Natick, MA) post-processing software from the University of Virginia [19–21]. The first stage of analysis was anatomical delineation, using either a contour or a region of interest covering the LV in the imaged slice. For long-axis images, this was done with a single line contour placed in the mesocardium, midway between the epicardium and endocardium (Fig. 2). For SAX images, both contour analysis and a region of interest were defined, with the region of interest manually defined between endo- and epicardial borders (Fig. 2). These contours were defined in either the peak systolic frame or a diastolic frame before automated propagation to the other frames (motion guided segmentation [22]), with subsequent manual adjustment as required. The frames with poor SNR at the end of cardiac cycle, where the myocardial outline could not be discerned, were discarded. Strain was then calculated in the segmented areas, generating regional polar-strain/time curves for radial and circumferential strain, contour strain/time curves for longitudinal strain in 2 planes and contour strain/time curves for short-axis strain. From these, global peak strain results were derived for each participant: SAX contour strain, longitudinal strain in the HLA and VLA planes, radial strain, and circumferential strain.

### Statistical analysis

Continuous data are expressed as mean ± standard deviation or median (inter-quartile range) and compared using the *t* test or Mann–Whitney test, respectively. Categorical data are expressed as number and percentages, and compared using Fisher’s exact test. Univariable linear regression was used to display the relationship between strain data and LVEF. Correlation testing using the Pearson correlation coefficient was performed to define the relationship between strain data and CMR parameters and visualised using the corrplot package in R. A random subset of studies (*n* = 5 controls and *n* = 5 DCM) was analysed by an independent operator and intraclass correlation coefficients were calculated to evaluate inter-observer variability. A *p* value of < 0.05 was considered significant and all analyses were conducted in the R statistical environment (version 3.3.1) or MATLAB (R2018a, pilot data only).

## Results

### Phantom study

**Fig. 3:**
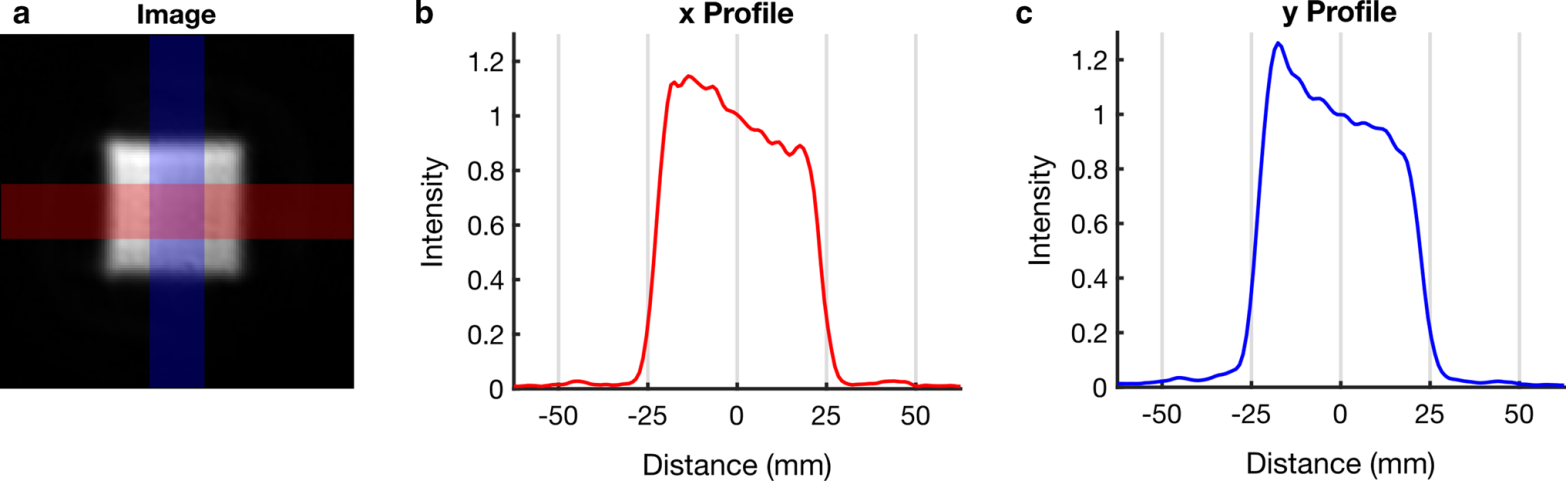
Measuring the in-plane excitation profile. Magnitude DENSE image acquired with a 50 mm stimulated echo FOV and a 250 mm readout FOV (cropped to 125 mm) (**a**). Excitation profiles, averaged over the red (**b**) and blue (**c**) regions shown in a. For the purposes of plotting intensity profiles, intensities were normalised to the intensity at the centre of the FOV. Profiles were measured from left to right (**b**) and from top to bottom (**c**). The receive coil spatial sensitivity variation causes the slope across the central region of the profiles. Receive coils were placed above (flexible matrix array) and below (spine coil, built into bed) the phantom (top and bottom of **a**) and sum of squares coil combination was used

Figure 3 shows the in-plane excitation profile. There is no visible signal outside the excited region and the full-width half maximum of the profile is 47 mm in both directions (prescribed size of 50 mm). Supplementary Fig. 1 shows that with an equal stimulated echo and readout FOV, there is no visible aliasing in the magnitude or phase images, but with increasing stimulated echo FOV, spiral aliasing becomes apparent.

The magnitude image SNR is reduced by 7% when using the stimulated echo FOV reduction method (270 ± 120 vs. 290 ± 100, for 20RR interval acquisition with and without the reduced stimulated echo FOV, respectively). Using the 14RR interval acquisition SNR (174 ± 69) is 36% lower than the 20RR interval acquisition (with reduced stimulated echo FOV) and 56% lower using the 8RR interval acquisition (SNR = 119 ± 53).

### Pilot in vivo data

Supplementary Fig. 2 plots peak radial strain for the three protocols acquired in the eight healthy volunteers. A positive bias is defined as an over-estimation relative to 20RR interval data, biases are quoted as median (inter-quartile range), and a Wilcoxon sign-rank test was used for comparison with the 20RR interval data. There are no significant differences and a small bias between the peak radial strains acquired with the 14RR intervals (*p* = 1.0, − 0.028 [0.13]) and the 8RR intervals (*p* = 0.74, − 8.8 × 10^−4^ [0.086]). There was also no significant bias in the peak circumferential strains acquired with the 8RR interval acquisition (*p* = 0.15, 0.013 [0.022]). There is a small (6%) but significant over-estimation in circumferential strain using the 14RR interval acquisition (*p* = 0.008, 0.0097 [0.0101]).

### Cohort demographics

In total, 47 participants were recruited to the study (DCM patients, *n* = 29; healthy controls, *n* = 18). Baseline characteristics are summarized in Table 1. Controls were younger than DCM patients (44.0 years vs. 52.8 years, *p* = 0.02). There was no significant difference in gender (DCM 72% male vs. controls 61% male, *p* = 0.52) or body surface area between patients and controls. DCM patients had higher indexed left ventricular (LV) volumes (both end diastolic and end systolic), higher indexed LV mass, lower LV stroke volume, and lower LVEF compared to healthy volunteers (Table 1).

**Table 1:** Baseline demographics and CMR findings

	DCM (*n* = 29)	Control (*n* = 18)	*p*
Age (years)	52.8 (12.0)	44.0 (11.3)	0.02
Gender = M	21 (72.4)	11 (61.1)	0.52
Body surface area (m^2^)	2.00 (0.26)	1.90 (0.22)	0.17
Left bundle branch block	6 (20.7)	0 (0.0)	0.07
Systolic blood pressure (mmHg)	123 (16)	109 (12)	0.002
Resting heart rate (bpm)	67 (15)	61 (8)	0.10
NYHA (%)			< 0.001
1	14 (48.3)	18 (100.0)	
2	14 (48.3)	0 (0.0)	
3	1 (3.4)	0 (0.0)	
LVEDVi (mL/m^2^)	122.7 (24.9)	84.8 (10.1)	< 0.001
LVESVi (mL/m^2^)	74.3 (23.8)	29.4 (6.0)	< 0.001
LVSVi (mL/m^2^)	48.5 (12.6)	55.3 (7.4)	0.04
LVMi (g/m^2^)	79.9 (23.5)	55.9 (20.7)	0.001
LVEF (%)	40.2 (10.4)	65.4 (5.2)	< 0.001
RVEDVi (mL/m^2^)	94.6 (22.0)	89.8 (12.9)	0.41
RVESVi (mL/m^2^)	48.2 (15.3)	37.8 (8.3)	0.01
RVSVi (mL/m^2^)	46.4 (12.3)	52.2 (8.3)	0.08
RVEF (%)	49.4 (9.1)	58.3 (5.8)	0.001
Maximum LV wall thickness (mm)	11.4 (2.2)	9.1 (1.7)	0.001
Mean lateral wall thickness (mm)	6.5 (1.4)	5.9 (1.2)	0.14
Mean septal wall thickness (mm)	9.1 (1.6)	7.1 (1.1)	< 0.001

Continuous data are shown as mean (± standard deviation) and compared using the *t* test or Mann–Whitney test; categorical data are shown as count (percentages) and compared using Fisher’s exact test

*LV/RV* left/right ventricular, *EF* ejection fraction, *EDVi/ESVi* indexed end diastolic/end systolic volume, *SVi* indexed stroke volume, *LVMi* indexed LV mass, *LAA/RAA* left/right atrial area, *NYHA* New York Heart Association class

### Feasibility of DENSE acquisition and assessment

**Fig. 4:**
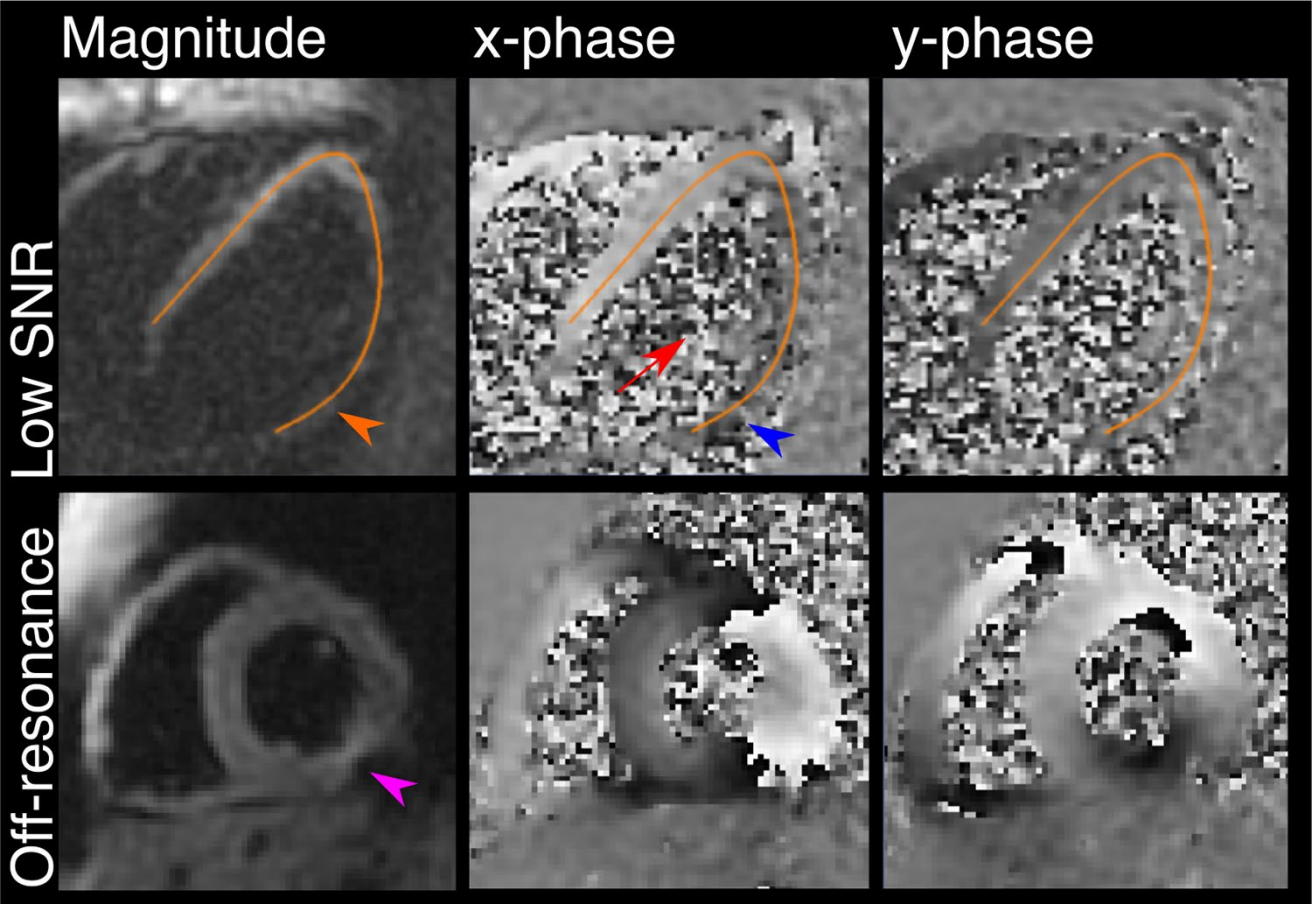
Cine DENSE analysis. Examples of studies in which cine DENSE data analysis was not possible due to insufficient signal-to-noise ratio (top panels) and off-resonance due to a change in magnetic susceptibility (bottom panels). The orange and blue arrows highlight the poor SNR in the lateral wall, which approaches background noise levels (red arrow). A susceptibility or off-resonance artefact often affects the short-axis data (purple arrow). The lower phase image shows phase wrap as the frames shown are acquired near peak systole. The DENSE processing software used here unwraps the phase

Cine DENSE data were successfully acquired in all participants. All DCM patients were able to complete the required breath hold duration. Elements of the data were not analysable in 1 control subject (6%) and 4 DCM patients (14%). In 1 control subject, the radial and circumferential strain data could not be analysed. In 1 DCM patient, no strain data were analysable. In 2 DCM patients, the radial and circumferential strain data could not be analysed. In 1 DCM patient, the VLA, radial and circumferential strain could not be analysed. Images were excluded from analysis because of insufficient SNR to permit reliable analysis or due to off-resonance artefacts (see Fig. 4).

### DENSE results

**Fig. 5:**
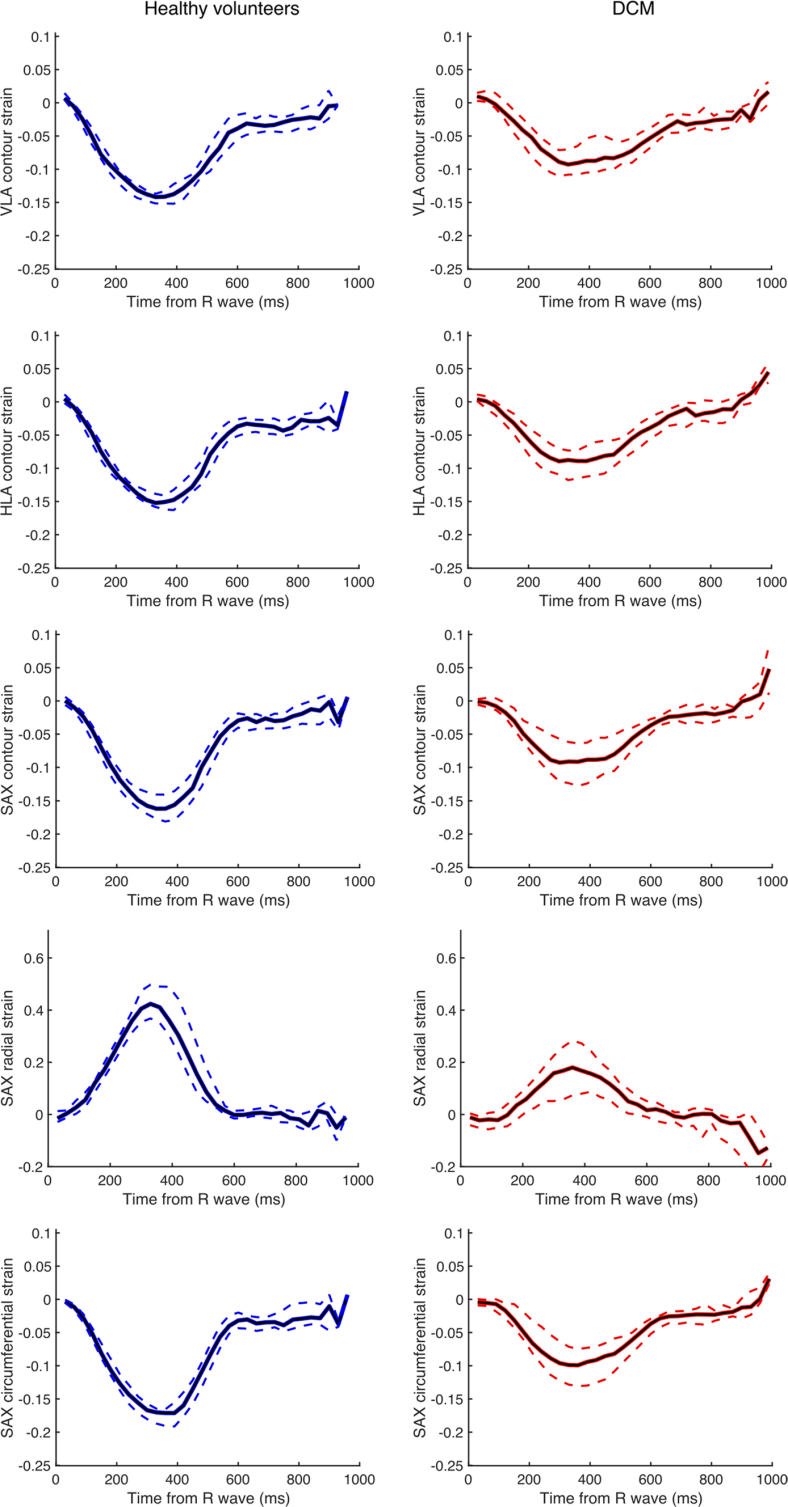
Summary strain–time curves for DCM and control cohort. The plots show the median longitudinal (horizontal long axis, HLA; vertical long axis, VLA) strain, short-axis (SAX) contour strain, and radial and circumferential strain in healthy volunteers (blue curves) and the DCM cohort (red curves). Curves are shown with the inter-quartile range (dashed lines). Peak strain is reduced in all dimensions in DCM patients compared to controls

Summary strain curves for the DCM and control cohorts are shown in Fig. 5. Compared to healthy volunteers, DCM patients had reduced peak global longitudinal (VLA and HLA) and short-axis strain (contour, radial and circumferential) (Table 2).

**Table 2:** Peak global long axis (HLA, VLA) and SAX strain in DCM patients and healthy volunteers (HVOL)

	DCM (*n* = 29)	HVOL (*n* = 18)	*p* value
VLA contour peak global strain	− 0.10 [− 0.11, − 0.06]	− 0.15 [− 0.16, − 0.14]	< 0.001
HLA contour peak global strain	− 0.09 [− 0.11, − 0.07]	− 0.16 [− 0.16, − 0.14]	< 0.001
SAX contour peak global strain	− 0.10 [− 0.12, − 0.07]	− 0.16 [− 0.18, − 0.14]	< 0.001
Radial peak global strain	0.18 [0.08, 0.28]	0.42 [0.37, 0.51]	< 0.001
Circumferential peak global strain	− 0.11 [− 0.13, − 0.08]	− 0.17 [− 0.19, − 0.17]	< 0.001

Data are shown as median (inter-quartile range)

Groups are compared using the Mann–Whitney test

### Inter-observer reliability

Reliability estimates between two observers are shown in Table 3. With the exception of radial strain, there is good inter-observer agreement in strain measurements (intraclass correlation coefficients > 0.80).

**Table 3:** Inter-observer reliability estimates

Strain	Observer 1	Observer 2	Absolute difference	ICC
VLA	− 0.13 (− 0.14, − 0.12)	− 0.13 (− 0.14, − 0.10)	0.02 (0.04)	0.83 (0.46 to 0.96)
HLA	− 0.12 (− 0.15, − 0.08)	− 0.14 (− 0.15, − 0.09)	0.02 (0.02)	0.97 (0.86 to 0.99)
SAX contour	− 0.14 (− 0.16, − 0.08)	− 0.14 (− 0.17, − 0.09)	0.02 (0.02)	0.89 (0.61 to 0.97)
Radial	0.36 (0.19, 0.44)	0.36 (− 0.15, 0.39)	0.16 (0.10)	0.75 (0.27 to 0.94)
Circumferential	− 0.15 (− 0.17, − 0.09)	− 0.16 (− 0.17, − 0.09)	0.02 (0.04)	0.84 (0.48 to 0.96)

Table shows the median (IQR) for strain measurements for both observers. The mean absolute difference (standard deviation) between the observers and the intraclass correlation coefficient (ICC, 95% confidence intervals) are shown

### Relationship between strain data and other CMR parameters

**Fig. 6:**
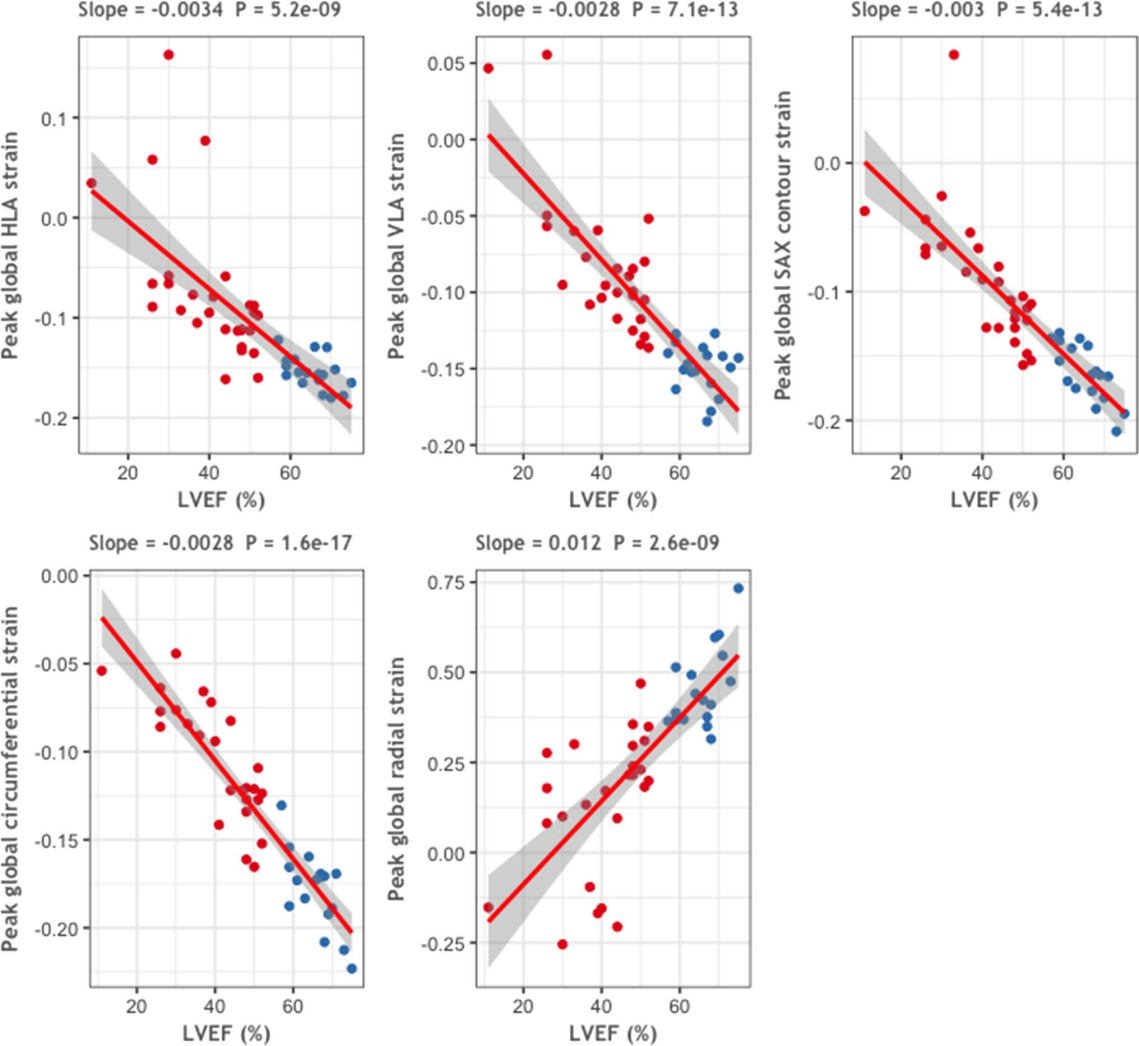
Relationship between LVEF and strain. Relationship between left ventricular ejection fraction (LVEF) and contour strains (top 3 panels) and radial and circumferential strains (bottom two panels). Univariable linear regression lines are plotted with 95% confidence intervals. The slope of the regression and the *p* value are shown above the plots. Points are colour coded by diagnosis; red = DCM, blue = healthy volunteer. The grey bands indicate 95% confidence intervals for the linear model

Across the cohort, there was a strong linear relationship between LVEF and contour strain and LVEF and circumferential strain in healthy volunteers and DCM patients (Fig. 6). There was also a linear relationship between LVEF and radial strain in healthy volunteers, though in DCM patients, this could not be clearly established, particularly for patients with severely impaired LVEF (Fig. 6).

**Fig. 7:**
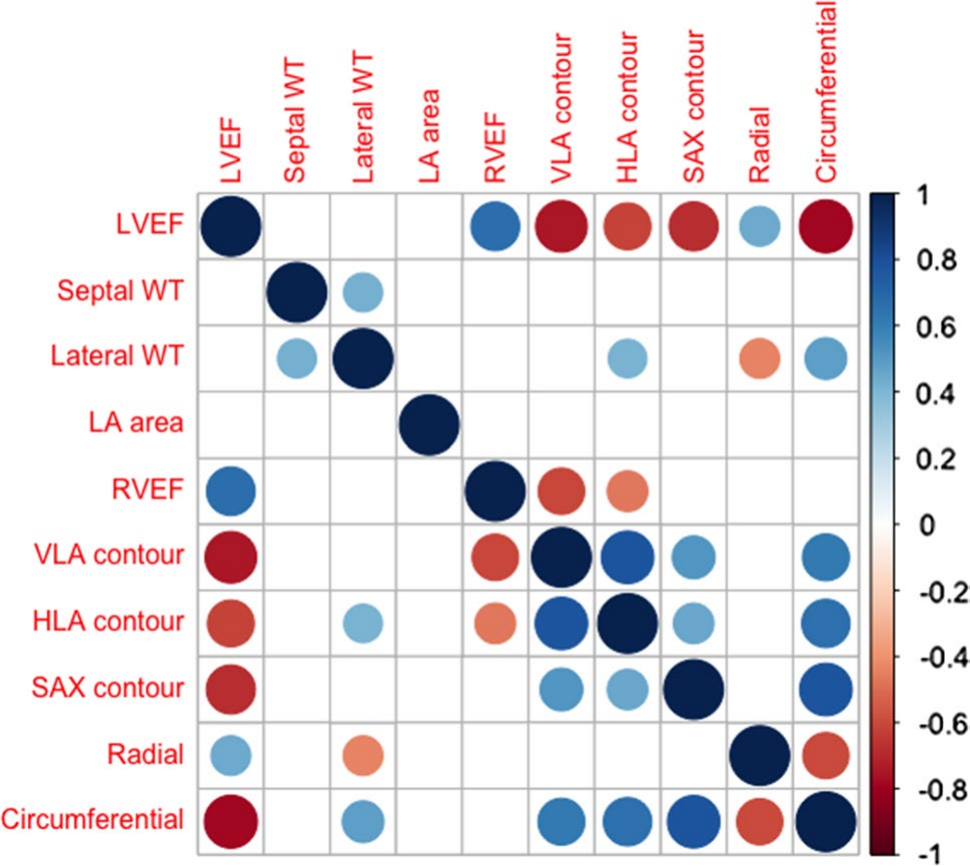
Correlation between strain and CMR parameters. Correlation matrix showing the size of the correlation (from − 1 to + 1) between LVEF, mean wall thickness (WT), left atrial (LA) area, right ventricular ejection fraction (RVEF) and strain, in DCM patients only. Only correlations with a *p* value < 0.05 are displayed. The larger the circle and the more intense colour indicate a stronger correlation. Overall, these results suggest that radial strain in this cohort is a less robust marker for the assessment of LVEF function compared to circumferential, longitudinal, and short-axis contour strain

The strength of correlation between measurements of strain and LVEF, mean septal and lateral wall thickness, and left atrial area in DCM patients only is shown in Fig. 7. This shows that LVEF is strongly negatively correlated with circumferential, VLA, HLA, and SAX contour strains and moderately positively correlated with radial strain. Lateral wall thickness is moderately correlated with radial, circumferential, and HLA contour strain estimates, whereas septal wall thickness is not correlated with strain in DCM patients. As expected, both horizontal and vertical long-axis estimates of longitudinal strain are strongly positively correlated and SAX contour strain is strongly positively correlated with circumferential strain in DCM patients. Right ventricular ejection fraction is weak–moderately correlated with longitudinal strain, but is not correlated with LV radial or circumferential strain. LA area is not correlated with any estimate of strain.

## Discussion

In this study, myocardial strain was assessed through the use of a novel cine DENSE sequence with a clinically feasible breath hold duration. We demonstrated a comprehensive strain assessment, including circumferential, radial, and longitudinal strain using this novel sequence in a cohort of healthy individuals and then established its ability to detect reduced strain in DCM patients. These findings are an important step towards the rapid clinical assessment of myocardial strain as part of a comprehensive CMR exam for DCM.

Although LVEF is the mainstay for assessment of cardiac function, it does not comprehensively assess myocardial function, is subject to high inter-observer variability, and is poorly discriminatory of sub-clinical disease [1]. Myocardial strain assessment may better reflect systolic function [2]. However, strain assessment in CMR has been limited by long scan times and complex post-processing.

Previous cine DENSE sequences have been shown to assess strain, but have been limited by long breath hold durations or unreliable navigator gated acquisitions. However, in this study, we made modifications to reduce the breath hold time to a clinically feasible duration for patients with DCM.

Initial phantom results show that the time-reversed pair of asymmetric RF pulses produce negligible residual aliasing artefact when the stimulated echo FOV is chosen to match the readout FOV. A stimulated echo FOV smaller than the readout FOV was not directly, evaluated, but no additional artefacts are expected within the stimulated echo FOV in this case. While the use of the reduced FOV method had a negligible effect on the SNR (7%), a reduction in acquisition duration and, therefore, FOV, resulted in a reduced SNR (36% reduction for the 14RR sequence in our phantom). In vivo, differences in peak strain between the techniques in the pilot study were small (bias was ≲ 8% of the mean). Based on these initial results and the FOV required for long-axis imaging, we selected the 14RR interval acquisition for evaluation in a cohort of DCM patients. The use of a spiral k-space trajectory limits the technique to a square FOV. Long-axis views may be more efficiently covered by a rectangle; however, we were able to image all healthy volunteers and patients within the 224 × 224 mm^2^ FOV acquired within our 14RR-interval breath hold.

On application to control subjects and DCM patients, all participants were able to complete the required breath hold. This demonstrates the feasibility of using a reduced breath hold cine DENSE CMR sequence for myocardial strain evaluation. This is a protocol that could be easily incorporated into a clinical exam.

As expected, DCM patients had reduced peak strain compared to controls, measured in both the long and short axes. Longitudinal strain and LVEF evaluate different aspects of myocardial deformation. While LVEF largely reflects radial contraction, longitudinal strain is a measure of the active shortening of the LV in the longitudinal direction and represents, amongst other factors, the function of longitudinally orientated cardiomyocytes in the sub-endocardium [23]. These cardiomyocytes are more sensitive to reduced coronary perfusion and increased wall stress; therefore, longitudinal dysfunction may be an early marker of LV dysfunction [4]. Longitudinal strain assessments could, therefore, be used to assess sub-clinical disease, for example, in genotype positive phenotype negative individuals. Longitudinal strain is also of prognostic importance in DCM [3]. LV longitudinal strain assessed from CMR via manual distance measurements [24] and feature tracking has also been shown to be an independent predictor of survival in DCM [25]. However, while feature tracking has provided many insights into myocardial function, it is hindered by the limited number of CMR visible features within the myocardium and errors may be introduced via through-plane motion [26]. DENSE has been shown to be more reproducible than feature tracking [27].

Therefore, a potential important strength of this technique that may have research and clinical utility is in the ability to assess longitudinal strain by CMR in DCM patients with minimal increase in exam duration. Indeed, myocardial strain appears to be a distinct CMR biomarker in this cohort. The left atrium has been identified as a CMR biomarker [28], but in this cohort, somewhat surprisingly, left atrial area was not correlated with any estimate of strain.

Although data acquisition was feasible in the majority of cases, complete analysis was not possible for a proportion of subjects (6% of healthy volunteers and 14% of patients), which is consistent with the previous literature, where failure rates are reported [27, 29]. This was due to insufficient SNR or off-resonance/susceptibility artefacts, which results in image blurring or distortion. We have not attempted to make SNR measurements in our in vivo data due to the difficulties with measuring SNR in magnitude images and the changing signal intensity in the myocardium and blood pool that is present with cine DENSE. Future studies should consider the effects of SNR on strain values derived from DENSE and the minimum SNR required for analysis, potentially using synthetic data. Off-resonance and susceptibility artefacts could be reduced using shorter duration spirals in combination with techniques such as parallel imaging (if SNR allows), compressed sensing or machine learning-based reconstructions. Alternatively, such acceleration techniques could be used to accelerate acquisitions with a full or reduced field of view, as the spiral trajectories used in this work were all designed to be fully sampled. Future studies should consider comparing DENSE acquisitions accelerated using the reduced field of view method and accelerated imaging methods [30].

Horizontal long-axis strain analysis was the most robust on inter-observer variability, compared to vertical long-axis strain. The analysis most susceptible to failure in both controls and patients was assessment of strain using endo and epicardial regions of interest in the SAX slice to determine circumferential and radial strain. This is due to both the limited spatial resolution and the thickness of the myocardial wall, which makes radial strain the most challenging measurement. In total, SAX ROI strain could not be assessed in five subjects, but of these, contour SAX strain assessment was possible in four subjects. SAX contour strain was also the most reproducible short-axis strain measurement. Across the DCM cohort, SAX contour strain showed strong correlation with circumferential strain, and radial strain measurements showed little correlation with LVEF. Radial strain measurements were the least reproducible between observers. Therefore, SAX contour strain may be a more robust measurement of global strain in DCM patients than ROI-defined SAX strain measures. As contour strain analysis is less time consuming compared to ROI analysis, this may facilitate real world clinical application of this technique.

The primary aim of this study was to demonstrate feasibility of assessment of strain in a control cohort and patient population using a modified cine DENSE sequence. However, reproducibility of the strain measures between centres will, to some extent, determine the diagnostic utility of DENSE derived strain measurements within this context, particularly given the relatively small changes in strain between DCM and healthy cohorts.

Only the 14RR-interval acquisition was performed in the DCM and full healthy cohort, as other protocols would have added long breath holds to a long protocol. As a consequence, it is not possible to assess whether the diagnostic performance of the DENSE sequence is altered by the use of the reduced FOV technique. However, in our initial pilot cohort, despite the reduction in SNR associated with the reduced FOV technique, the peak strain values were similar between the shortened and full DENSE acquisitions. Future studies may consider adapting the FOV and hence the breath hold duration based on the patient anatomy and scan plane.

In conclusion, this study demonstrates the feasibility of CMR assessment of myocardial strain in DCM patients and healthy controls using a novel cine DENSE technique which enabled a reduction in breath hold duration by one-third. This may facilitate the integration of myocardial strain assessment into routine CMR studies, providing a one stop imaging investigation for patients with DCM.

## Electronic supplementary material

Below is the link to the electronic supplementary material.

Supplementary material 1 (DOCX 3949 kb)

## Abbreviations

DCM: Dilated cardiomyopathy
CMR: Cardiovascular magnetic resonance
DENSE: Displacement encoding with stimulated echoes
HLA: Horizontal long axis
VLA: Vertical long axis
SAX: Short axis
LVEF: Left ventricular ejection fraction
FOV: Field of view
SNR: Signal-to-noise ratio

## References

[1] Januzzi JL, Chandrashekhar Y (2017). Strain echocardiography: the new gold standard for imaging ventricular function?. J Am Coll Cardiol.

[2] Stokke TM, Hasselberg NE, Smedsrud MK, Sarvari SI, Haugaa KH, Smiseth OA, Edvardsen T, Remme EW (2017). Geometry as a confounder when assessing ventricular systolic function: comparison between ejection fraction and strain. J Am Coll Cardiol.

[3] Chimura M, Onishi T, Tsukishiro Y, Sawada T, Kiuchi K, Shimane A, Okajima K, Yamada S, Taniguchi Y, Yasaka Y, Kawai H (2017). Longitudinal strain combined with delayed-enhancement magnetic resonance improves risk stratification in patients with dilated cardiomyopathy. Heart.

[4] Buckberg G, Hoffman JI, Mahajan A, Saleh S, Coghlan C (2008). Cardiac mechanics revisited: the relationship of cardiac architecture to ventricular function. Circulation.

[5] Gulati A, Jabbour A, Ismail TF, Guha K, Khwaja J, Raza S, Morarji K, Brown TD, Ismail NA, Dweck MR, Di Pietro E, Roughton M, Wage R, Daryani Y, O’Hanlon R, Sheppard MN, Alpendurada F, Lyon AR, Cook SA, Cowie MR, Assomull RG, Pennell DJ, Prasad SK (2013). Association of fibrosis with mortality and sudden cardiac death in patients with nonischemic dilated cardiomyopathy. JAMA.

[6] Puntmann VO, Carr-White G, Jabbour A, Yu CY, Gebker R, Kelle S, Hinojar R, Doltra A, Varma N, Child N, Rogers T, Suna G, Arroyo Ucar E, Goodman B, Khan S, Dabir D, Herrmann E, Zeiher AM, Nagel E (2016). T1-mapping and outcome in nonischemic cardiomyopathy: all-cause mortality and heart failure. JACC Cardiovasc Imaging.

[7] Zerhouni EA, Parish DM, Rogers WJ, Yang A, Shapiro EP (1988). Human heart: tagging with MR imaging–a method for noninvasive assessment of myocardial motion. Radiology.

[8] Axel L, Dougherty L (1989). MR imaging of motion with spatial modulation of magnetization. Radiology.

[9] Simpson RM, Keegan J, Firmin DN (2013). MR assessment of regional myocardial mechanics. J Magn Reson Imaging: JMRI.

[10] Aletras AH, Ding S, Balaban RS, Wen H (1999). DENSE: displacement encoding with stimulated echoes in cardiac functional MRI. J Magn Reson (San Diego, Calif: 1997).

[11] Wedeen VJ, Weisskoff RM, Reese TG, Beache GM, Poncelet BP, Rosen BR, Dinsmore RE (1995). Motionless movies of myocardial strain-rates using stimulated echoes. Magn Reson Med.

[12] Kim D, Gilson WD, Kramer CM, Epstein FH (2004). Myocardial tissue tracking with two-dimensional cine displacement-encoded MR imaging: development and initial evaluation. Radiology.

[13] Taylor AM, Jhooti P, Wiesmann F, Keegan J, Firmin DN, Pennell DJ (1997). MR navigator-echo monitoring of temporal changes in diaphragm position: implications for MR coronary angiography. J Magn Reson Imaging: JMRI.

[14] Zhong X, Spottiswoode BS, Meyer CH, Kramer CM, Epstein FH (2010). Imaging three-dimensional myocardial mechanics using navigator-gated volumetric spiral cine DENSE MRI. Magn Reson Med.

[15] Reeder SB, Wintersperger BJ, Dietrich O, Lanz T, Greiser A, Reiser MF, Glazer GM, Schoenberg SO (2005). Practical approaches to the evaluation of signal-to-noise ratio performance with parallel imaging: application with cardiac imaging and a 32-channel cardiac coil. Magn Reson Med.

[16] Scott AD, Tayal U, Nielles-Vallespin S, Ferreira P, Zhong X, Epstein FH, Prasad SK, Firmin D (2016). Accelerating cine DENSE using a zonal excitation. J Cardiovasc Magn Reson.

[17] Shah S, Kellman P, Greiser A, Weale P, Zuehlsdorff S, Jerecic R (2009). Rapid fieldmap estimation for cardiac shimming. Proc Intl Soc Mag Reson Med.

[18] Maceira AM, Prasad SK, Khan M, Pennell DJ (2006). Normalized left ventricular systolic and diastolic function by steady state free precession cardiovascular magnetic resonance. J Cardiovasc Magn Reson.

[19] Gilliam AD, Epstein FH (2012). Automated motion estimation for 2-D cine DENSE MRI. IEEE Trans Med Imaging.

[20] Spottiswoode BS, Zhong X, Hess AT, Kramer CM, Meintjes EM, Mayosi BM, Epstein FH (2007). Tracking myocardial motion from cine DENSE images using spatiotemporal phase unwrapping and temporal fitting. IEEE Trans Med Imaging.

[21] Gilliam AD, Suever JD (2016) DENSE analysis: cine DENSE processing software. https://github.com/denseanalysis/denseanalysis. Accessed 26 Jul 2017

[22] Spottiswoode BS, Zhong X, Lorenz CH, Mayosi BM, Meintjes EM, Epstein FH (2009). Motion-guided segmentation for cine DENSE MRI. Med Image Anal.

[23] Stanton T, Marwick TH (2010). Assessment of subendocardial structure and function. JACC: Cardiovasc Imaging.

[24] Riffel JH, Keller MG, Rost F, Arenja N, Andre F, Aus dem Siepen F, Fritz T, Ehlermann P, Taeger T, Frankenstein L, Meder B, Katus HA, Buss SJ (2016). Left ventricular long axis strain: a new prognosticator in non-ischemic dilated cardiomyopathy?. J Cardiovasc Magn Reson.

[25] Buss SJ, Breuninger K, Lehrke S, Voss A, Galuschky C, Lossnitzer D, Andre F, Ehlermann P, Franke J, Taeger T, Frankenstein L, Steen H, Meder B, Giannitsis E, Katus HA, Korosoglou G (2015). Assessment of myocardial deformation with cardiac magnetic resonance strain imaging improves risk stratification in patients with dilated cardiomyopathy. Eur Heart J Cardiovasc Imaging.

[26] Cowan BR, Peereboom SM, Greiser A, Guehring J, Young AA (2015). Image Feature Determinants of Global and Segmental Circumferential Ventricular Strain From Cine CMR. JACC Cardiovasc Imaging.

[27] Mangion K, Gao H, McComb C, Carrick D, Clerfond G, Zhong X, Luo X, Haig CE, Berry C (2016). A novel method for estimating myocardial strain: assessment of deformation tracking against reference magnetic resonance methods in healthy volunteers. Sci Rep.

[28] Wijesurendra RS, Rider OJ, Neubauer S (2017). Left atrial volumes in health and disease measured using cardiac magnetic resonance. Circ Cardiovasc Imaging.

[29] Nielles-Vallespin S, Khalique Z, Ferreira PF, de Silva R, Scott AD, Kilner P, McGill LA, Giannakidis A, Gatehouse PD, Ennis D, Aliotta E, Al-Khalil M, Kellman P, Mazilu D, Balaban RS, Firmin DN, Arai AE, Pennell DJ (2017). Assessment of myocardial microstructural dynamics by in vivo diffusion tensor cardiac magnetic resonance. J Am Coll Cardiol.

[30] Chen X, Yang Y, Cai X, Auger DA, Meyer CH, Salerno M, Epstein FH (2016). Accelerated two-dimensional cine DENSE cardiovascular magnetic resonance using compressed sensing and parallel imaging. J Cardiovasc Magn Reson.

