# Supplementary material for: The feasibility of a novel limited field of view spiral cine DENSE sequence to assess myocardial strain in dilated cardiomyopathy
Source: MAGMA. 2019 Jan 29;32(3):317–29. doi: 10.1007/s10334-019-00735-5 (PMC6525145; doi:10.1007/s10334-019-00735-5)
Supplement: Supplementary file 1 — Supplementary material 1 (DOCX 3949 kb) [file 10334_2019_735_MOESM1_ESM.docx]

# THE FEASIBILITY OF A NOVEL LIMITED FIELD OF VIEW SPIRAL CINE DENSE SEQUENCE TO ASSESS MYOCARDIAL STRAIN IN DILATED CARDIOMYOPATHY

# Supplementary material

Diagnosis of DCM

The diagnosis of DCM was made based on CMR evidence of left ventricular dilation and systolic impairment with reference to age, gender, and body surface area adjusted nomograms(1). Exclusion criteria for DCM included a history of uncontrolled systemic hypertension, coronary artery disease (>50% stenosis in one or more major epicardial arteries or previous percutaneous coronary intervention or coronary artery bypass grafting), chronic excess alcohol consumption meeting criteria for alcoholic cardiomyopathy (>80g/day for more than 5 years(2)), systemic disease known to cause DCM, pericardial disease, congenital heart disease, or significant primary valvular disease(3-5).

**Acquisition and assessment of cardiac structure and function**

End-expiratory breath hold balanced steady state free precession (bSSFP) cine images were acquired in the 3 long axis planes (horizontal long axis, HLA; right ventricular outflow tract, RVOT; left ventricular outflow tract, LVOT) and 8mm short axis slices (2mm gap) from the atrioventricular ring to the apex ([5](#_ENREF_5)).

Typical acquisition parameters were: acquired resolution 1.5x1.5mm, TR/TE 2.8/1.2ms, FOV 340x240mm^2^, flip angle 44 degrees, GRAPPA parallel imaging acceleration factor x2, acquired temporal resolution 54ms and 25 reconstructed frames.

Biventricular volumes, function and left ventricular (LV) mass were measured using a semi-automated threshold-based technique (CMRtools, Cardiovascular Imaging Solutions, London, UK). All volume and mass measurements were indexed to body surface area and referenced to age and gender([17](#_ENREF_17)).

Septal and lateral LV wall thickness was measured at the level of the papillary muscles, mid cavity in end-diastole. An average of at least 2 measurements was taken. Maximum left ventricular wall thickness was measured in each short axis slice at end-diastole, with the exclusion of papillary muscles. The left atrial (LA) endocardial border was traced to determine LA area with exclusion of the pulmonary veins, LA appendage, and mitral valve recess. An average of two measurements was recorded.

**References**

1. Maceira AM, Prasad SK, Khan M, Pennell DJ. Normalized left ventricular systolic and diastolic function by steady state free precession cardiovascular magnetic resonance. J Cardiovasc Magn Reson 2006;8:417-426.

2. Guzzo-Merello G, Segovia J, Dominguez F et al. Natural history and prognostic factors in alcoholic cardiomyopathy. JACC Heart Fail 2015;3:78-86.

3. Elliott P. Cardiomyopathy. Diagnosis and management of dilated cardiomyopathy. Heart 2000;84:106-112.

4. Elliott P, Andersson B, Arbustini E et al. Classification of the cardiomyopathies: a position statement from the european society of cardiology working group on myocardial and pericardial diseases. European Heart Journal 2008;29:270-276.

5. Rapezzi C, Arbustini E, Caforio AL et al. Diagnostic work-up in cardiomyopathies: bridging the gap between clinical phenotypes and final diagnosis. A position statement from the ESC Working Group on Myocardial and Pericardial Diseases. Eur Heart J 2013;34:1448-1458.

**Supplementary figures**

Supplementary Figure 1: Assessing aliasing with an increasing ratio of stimulated echo FOV to readout FOV for a fixed readout FOV of 70x70mm^2^.


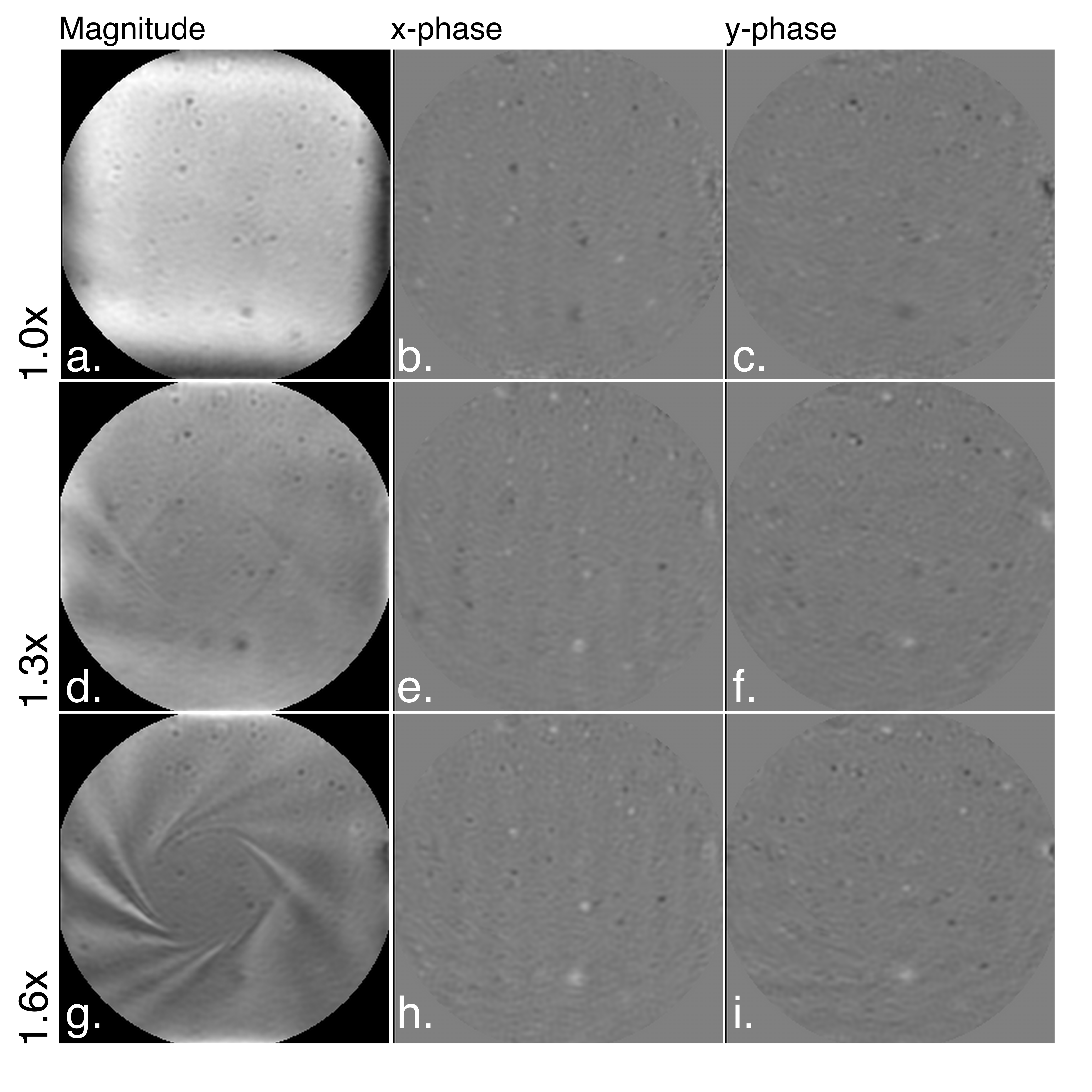


When the stimulated echo FOV and readout FOV are equal (top line), there is no aliasing artefact (a, b and c). As the stimulated echo FOV increases to 1.3x the readout FOV (d, e and f) and then 1.6x (g, h and i), there is increasing aliasing artefact. Images are masked to the diameter of the readout FOV.

Supplementary Figure 2: Results of the pilot study.


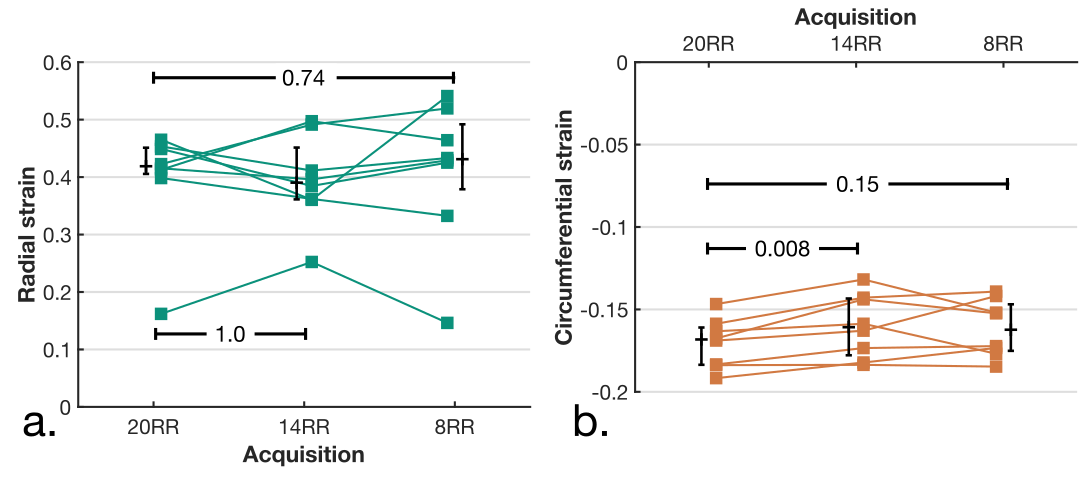


A comparison of three DENSE protocols acquired in 8 normal subjects in the pilot study for radial strain (a) and circumferential strain (b). The 3 protocols were acquired with 20RR-intervals, 14RR-intervals and 8RR-intervals as described in the methods.
